# Acute vascular redox modulation by SGLT2 inhibition in non-diabetic patients

**DOI:** 10.1186/s40842-026-00304-5

**Published:** 2026-06-18

**Authors:** Katica Cvitkusic Lukenda, Ana Cipak Gasparovic, Mirta Milic, Barbara Radovani, Frano Vuckovic, Jelena Jakab, Domagoj Vucic, Ana Kovacevic, Ivan Gudelj

**Affiliations:** 1 Department of Cardiology, General Hospital Dr. Josip Bencevic, A. Stampara 42, Slavonski Brod, HR-35000 Croatia; 2 Faculty of Dental Medicine and Health Osijek, Josip Juraj Strossmayer University of Osijek, Crkvena 21, Osijek, 31000 Croatia; 3 Rudjer Boskovic Institute, Bijenicka Cesta 54, Zagreb, 10000 Croatia; 4 Institute for Medical Research and Occupational Health, Ksaverska 2, Zagreb, 10000 Croatia; 5 Faculty of Biotechnology and Drug Development, University of Rijeka, Radmile Matejcic Street, Rijeka, 51000 Croatia; 6 Genos Glycoscience Research Laboratory, Borongojska 83H, Zagreb, 10000 Croatia; 7 Faculty of Pharmacy and Biochemistry, University of Zagreb, Ante Kovacica 1, Zagreb, 10000 Croatia

**Keywords:** Empagliflozin, Coronary angiography, Oxidative stress, Oxidative DNA damage, N-Glycosylation, Inflammation

## Abstract

**Background:**

Coronary angiography induces oxidative stress through contrast media exposure and ionizing radiation, potentially contributing to vascular and renal injury. Sodium-glucose cotransporter 2 inhibitors (SGLT2i) exert antioxidant and anti-inflammatory effects beyond glycemic control. We investigated whether a single pre-procedural dose of empagliflozin modulates oxidative stress and inflammatory glycosylation patterns in non-diabetic patients undergoing elective coronary angiography.

**Methods:**

In this prospective, randomized, double-blind study, 60 patients undergoing elective coronary angiography were assigned to standard care or empagliflozin 10 mg administered 2 h before the procedure. Blood samples were collected at baseline, 4 h, and 24 h post-procedure. Total antioxidant capacity (TAC), oxidative DNA damage (alkaline comet assay), and N-glycosylation profiles of immunoglobulin G (IgG) and total plasma proteins were analyzed. Longitudinal changes were assessed using mixed-effects models with correction for multiple testing.

**Results:**

Baseline characteristics and procedural variables were comparable between groups. Empagliflozin administration was associated with attenuation of oxidative DNA damage 24 h after angiography and stabilization of antioxidant capacity compared with standard care. Directional shifts in IgG N-glycosylation toward a less pro-inflammatory profile were observed in the intervention group, including reduced agalactosylated and core-fucosylated glycans and relative preservation of galactosylated structures. Similar modulatory trends were detected in total plasma protein glycosylation patterns. Although several glycomic changes did not reach statistical significance after correction for multiple testing, the overall biological signal consistently favored reduced oxidative and inflammatory activation in the empagliflozin group.

**Conclusions:**

A single pre-procedural dose of empagliflozin was associated with attenuation of oxidative stress-related DNA damage and modulation of inflammatory glycosylation patterns following coronary angiography. These findings suggest a potential peri-procedural cytoprotective role of SGLT2 inhibition that warrants confirmation in larger studies.

**Trial registration:**

ISRCTN11022820. Registered 13 October 2025. Retrospectively registered.

**Graphical Abstract:**

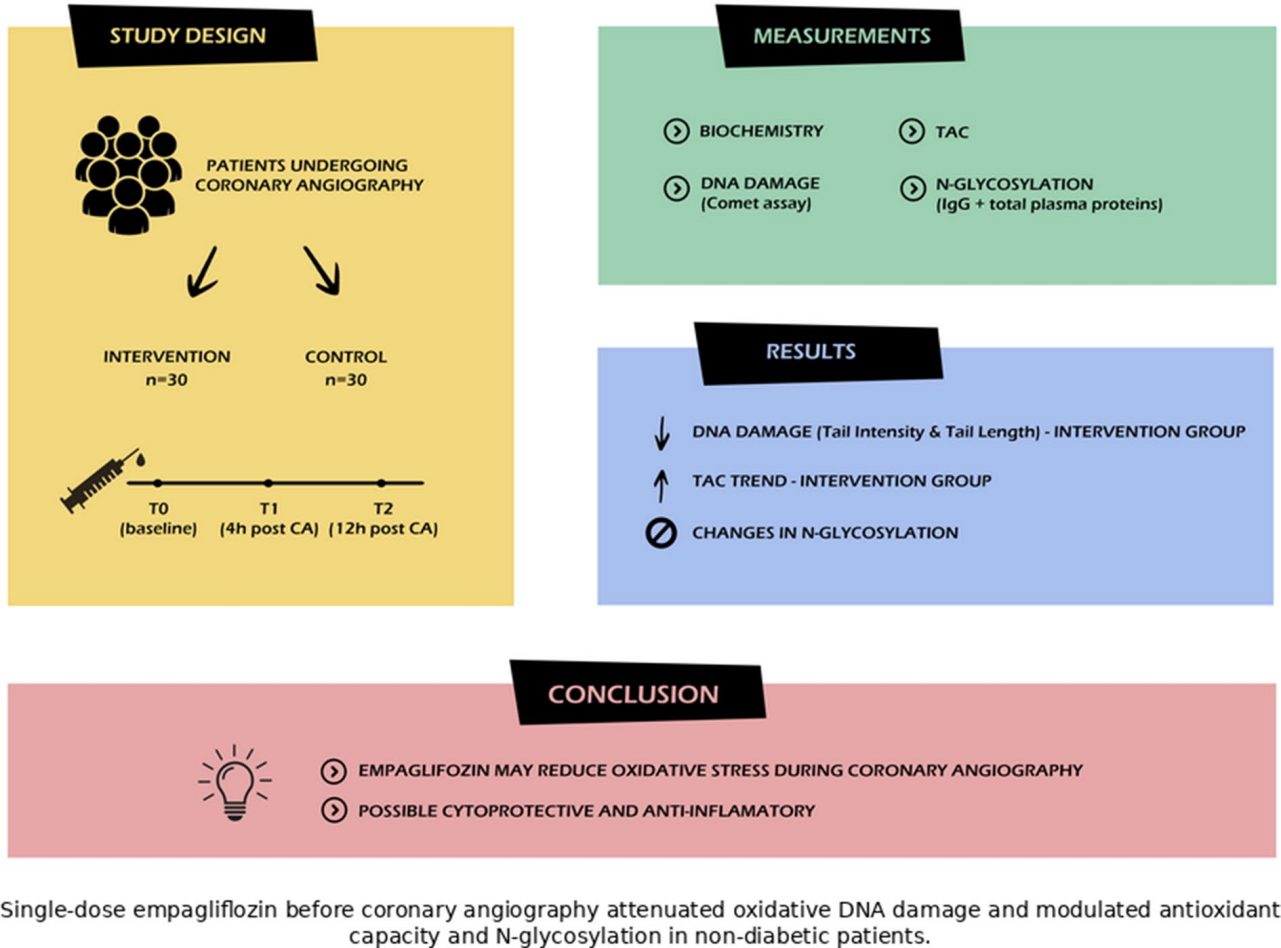

**Supplementary Information:**

The online version contains supplementary material available at 10.1186/s40842-026-00304-5.

## Condensed abstract

Coronary angiography induces transient oxidative and inflammatory responses that may contribute to vascular stress. In this randomized study of 60 patients, a single pre-procedural dose of empagliflozin was associated with attenuation of oxidative DNA damage and differential modulation of antioxidant capacity and N-glycosylation profiles. These findings suggest potential peri-procedural cytoprotective effects of SGLT2 inhibition during invasive cardiology procedures. Larger studies are required to determine the clinical relevance of these biomarker changes.

**Supplementary Information:**

The online version contains supplementary material available at 10.1186/s40842-026-00304-5.

## Background

Coronary angiography remains the gold standard for diagnosing coronary artery disease and involves exposure to iodine-based contrast agents and ionizing radiation [1]. Although generally safe, the procedure may induce oxidative stress through contrast-mediated cytotoxicity and radiation-induced generation of reactive oxygen species (ROS), contributing to transient renal and systemic injury [2, 3].

Oxidative stress disrupts the balance between ROS production, nitric oxide bioavailability, and endogenous antioxidant defenses, leading to endothelial dysfunction and impaired vasomotor, anti-inflammatory, and antithrombotic properties [4–7]. The endothelial glycocalyx, enriched with glycoproteins and N-glycans, plays a key role in vascular homeostasis and cellular signaling [8, 9]. Alterations in glycosylation patterns have been linked to inflammatory activation and cardiovascular disease [10, 11].

Sodium-glucose cotransporter 2 inhibitors (SGLT2i) are established cardiometabolic agents that reduce cardiovascular events and preserve renal function beyond glucose lowering [12–14]. Experimental and clinical studies indicate that SGLT2 inhibition attenuates oxidative stress and modulates inflammatory signaling pathways, partly through reductions in ROS production and enhancement of antioxidant defenses [15].

Given the acute redox and inflammatory perturbations induced by coronary angiography, we hypothesized that a single pre-procedural dose of empagliflozin could modulate oxidative stress and related inflammatory biomarkers.

## Methods

PARTICIPANTS. The study included 60 patients referred for elective coronary angiography at the General Hospital Dr. Josip Benčević in Slavonski Brod from October 2023 to January 2024. This prospective, double-blind study used computer-generated simple randomization (1:1) to assign participants to the control group (standard care) or the intervention group (standard care plus empagliflozin 10 mg two hours before coronary angiography). Randomization was performed using a computer-generated allocation sequence prepared by an independent investigator not involved in patient recruitment or outcome assessment. Allocation concealment was ensured using sequentially numbered opaque sealed envelopes opened only after participant enrollment. Both participants and investigators responsible for clinical evaluation, biomarker measurement, and statistical analysis were blinded to treatment allocation throughout the study. Standard care included measuring vital parameters and ECG, venous line placement, and administering 5 mg oral diazepam before elective coronary angiography via transradial or transfemoral access. Exclusion criteria: eGFR < 30 ml/min/1.73 m², NYHA IV heart failure, recent ACS/PCI (< 6 months), active malignancy, inflammatory or autoimmune disease, chronic SGLT2i use, diabetes, HFrEF, life expectancy < 12 months. A custom-designed questionnaire collected data on habits, menstrual history (for women), medical history, and chronic medications before sample collection. The primary outcome was procedure-related oxidative stress, assessed through complementary biomarkers including oxidative DNA damage (comet assay), total antioxidant capacity (TAC), and plasma N-glycan profiles. Secondary outcomes included renal, cardiac, and inflammatory markers (creatinine, eGFR, hs-troponin I, and hs-CRP). Harms were systematically monitored during and 24 h after coronary angiography.

BLOOD SAMPLING. Venous blood and plasma were collected at three time points: baseline (t0), 4 h ± 15 min (t1), and 24 h ± 15 min (t2) post-angiography. At t0, hematological and biochemical parameters, TAC, N-glycans (IgG and plasma proteins), and the comet assay were performed. At t1, TAC and comet assay were repeated, and at t2, creatinine, eGFR, hsCRP, hsTnI, TAC, N-glycans, and comet assay were assessed. Samples were immediately stored at − 80 °C after collection and kept until analysis for TAC assessment, IgG and total plasma proteins N-glycans, and for the comet assay.

TOTAL ANTIOXIDANT CAPACITY. The measurement of TAC is based on the reaction of endogenous antioxidants with hydrogen peroxide [16]. Briefly, the assay is based on the ability of endogenous antioxidants in plasma to reduce hydrogen peroxide. The remaining hydrogen peroxide reacts with peroxidase and tetramethylbenzidine (TMB), and the reaction is stopped with a stop solution (2 M H₂SO₄), changing the color from blue to yellow. The color intensity is measured spectrophotometrically using a microplate reader (EZ2000 Read, Biochrom, Cambridge, UK) at 450 nm. TAC values are expressed as uric acid equivalents (µM), with higher values indicating higher antioxidant capacity of the sample. The precision of the Total Antioxidant Capacity (TAC) assay was evaluated by calculating the intra- and inter-assay variability. The mean intra-assay coefficient of variation (CV) was 4.15%, with a median CV of 3.03%. The inter-assay mean CV was 3.09%, with a median CV of 3.12%.

IMMUNOGLOBULIN G AND TOTAL PLASMA PROTEIN N-GLYCANS. N-glycan analyses were performed on plasma samples according to the previously described protocol [17]. IgG was isolated by affinity chromatography, followed by denaturation, enzymatic release of N-glycans, and fluorescent labeling with 2-aminobenzamide (2-AB). Labeled N-glycans were analyzed using ultra-high-performance liquid chromatography (UHPLC).

Chromatograms were separated into 39 peaks corresponding to total plasma protein N-glycans and 24 peaks corresponding to IgG N-glycans. The relative abundance of each glycan peak was expressed as a percentage of the total integrated chromatogram area (Individual GP / Total GP × 100).

Derived glycan traits were calculated to reflect structural features. Agalactosylation (G0) included all complex N-glycan structures lacking galactose; monogalactosylation (G1) and digalactosylation (G2) represented structures with one or two galactose residues, respectively. Glycans containing bisecting N-acetylglucosamine were classified as bisecting (B), those containing core fucose as core fucosylation (CF), and glycans containing sialic acid as sialylation (S). Mathematical definitions of derived traits are provided in Supplemental Table 1.

Glycan peaks were assigned based on retention times and comparison with established glycan libraries. Chromatographic peak integration and quantification were performed using dedicated UHPLC analysis software. Quality control procedures included monitoring chromatographic consistency across analytical batches and evaluation of technical replicates to ensure analytical reliability. All glycan analyses were performed according to previously validated analytical protocols at the Genos Glycoscience Research Laboratory.

COMET ASSAY. The alkaline comet assay was performed according to the protocol described by Milić et al. [18]. Briefly, peripheral blood cells were embedded in agarose on microscope slides, lysed, and subjected to alkaline electrophoresis to allow migration of fragmented DNA.

Slides were analyzed using a fluorescence microscope (Olympus BX51, Tokyo, Japan) at 200× magnification equipped with a CCD camera and Comet Assay IV software (Instem, London, UK). The excitation filter range was 515–560 nm and the barrier filter was 590 nm. Illumination was provided by a LED light source (SET-COOL-0003 pE-300, CoolLED Ltd., Andover, UK).

For each gel, a minimum of 50 individual cells (“comets”) were manually evaluated. Two gels were analyzed per sample, resulting in at least 100 analyzed comets per participant. The primary parameter used for further analysis was tail intensity (TI), defined as the percentage of DNA in the comet tail, reflecting the extent of DNA damage. Tail length (TL), representing the migration distance of fragmented DNA during electrophoresis, was recorded.

Comet assay parameters were summarized using descriptive statistics including mean, median, minimum, maximum, standard deviation, and standard error. Replicate gels from the same sample showed no significant differences (compared with Breakdown ANOVA analysis after the logarithmic transformation of the data). The precision of the COMET assay was evaluated by calculating the intra- and inter-assay variability. The mean intra-assay coefficient of variation (CV) was 5%, while the inter-assay mean CV was 13%, with similar median values.

STATISTICAL METHODS. Data distribution was assessed using the Shapiro–Wilk test. Appropriate parametric or non-parametric tests were used for group comparisons and correlations. Longitudinal changes in glycans, comet assay parameters (tail intensity and tail length), and biochemical markers were analyzed using linear mixed-effects models adjusted for treatment group (empagliflozin vs. control). For glycan analysis, UHPLC peak areas were normalized to total chromatogram area, log-transformed, and batch-corrected using the ComBat method. To allow comparability of effect sizes across traits, variables were further normalized using inverse rank-based transformation. To account for multiple testing across glycan traits, false discovery rate (FDR) correction was applied using the Benjamini–Hochberg procedure, and adjusted p-values (p_adj) were used for interpretation of statistical significance in glycan analyses. Statistical significance was defined as *p* < 0.05 unless otherwise specified after multiple-testing correction. All analyses were performed in R (version 4.3.3). Comet assay results were compared with a validated reference database [18, 19]. Detailed statistical procedures are provided in the Supplementary Materials.

## Results

BASELINE AND PROCEDURAL CHARACTERISTICS. Baseline demographic, clinical, and laboratory characteristics were comparable between the control and intervention groups (Table 1; Supplemental Table S2). No significant differences were observed in age, anthropometric parameters, medical history, baseline medication use, or cardiac rhythm. Systolic blood pressure was modestly higher in the intervention group at baseline (Table 2), while other baseline variables were balanced between groups.

**Table 1:** General and clinical characteristics of participants

	Number (%) of participants			P*
	Control group	Intervention group	Total	
**Gender**				
Male (M)	15 (50)	15 (50)	30 (50)	> 0.99
Female (F)	15 (50)	15 (50)	30 (50)	
**Smoking and Alcohol**				
Smoking	12 (40)	8 (27)	20 (33)	0.27
Alcohol consumption	5 (17)	7 (23)	12 (20)	0.52
**Menopause (only females)**	13/15 (87)	14/15 (93)	27/30 (90)	> 0.99
**Medical History**				
MI	5 (17)	4 (13)	9 (15)	> 0.99^†^
PCI	4 (13)	3 (10)	7 (12)	> 0.99^†^
CVI	2 (7)	4 (13)	6 (10)	0.67^†^
**Therapies**				
Warfarin	1 (3)	1 (3)	2 (3)	> 0.99^†^
NOACs	3 (10)	2 (7)	5 (8)	> 0.99^†^
ASA	16 (53)	13 (43)	29 (48)	0.61
Antihypertensives	28 (93)	24 (80)	52 (87)	0.25^†^
RAASi	23 (82)	23 (96)	46 (89)	0.20
CCB	15 (54)	14 (58)	29 (56)	0.73
BB	22 (79)	13 (54)	35 (67)	0.06
diuretics	11 (39)	11 (45)	22 (42)	0.63
** Lipid-lowering Therapies**				
Statins	23 (77)	23 (77)	46 (77)	> 0.99
Ezetimibe	1 (3)	5 (17)	6 (10)	0.20^†^
Anti-PCSK9	1 (3)	0	1 (2)	> 0.99^†^
**Heart Rhythm**				
Sinus rhythm	28 (93)	29 (97)	57 (95)	> 0.99^†^
Atrial fibrillation	2 (7)	1 (3)	3 (5)	> 0.99^†^

ASA = acetylsalicylic acid; BB = beta-blockers; CCB = calcium channel blockers; CVI = cerebrovascular insult; MI = myocardial infarction; NOACs = new oral anticoagulants; PCI = percutaneous coronary intervention; RAASi = renin–angiotensin–aldosterone system inhibitors

*χ² test unless otherwise indicated; †Fisher’s exact test

**Table 2:** Baseline demographic and anthropometric characteristics between groups

	Control Median (IQR)	Intervention Median (IQR)	Difference	95% CI	P*
Age (years)	65 (59–68)	65 (60–72)	2	-3 to 6	0.49
Body weight (kg)	81.5 (72–85.3)	77 (70–95.3)	1	-8 to 10	0.85
Body height (cm)	167 (162–172)	165 (161–174)	-1	-6 to 4	0.67
BMI (kg/m^2^)	28.6 (25.3–31.5)	30.3 (26.3–32)	0.9	-1.5 to 3.3	0.42
WC (cm)	97.5 (92–104.3)	101 (89.8–107.3)	2	-4 to 8	0.53
SBP (mmHg)	124 (115–133)	133 (122–143)	9	1.5 to 16.5	0.02
DBP (mmHg)	76 (70–83)	79 (74–85)	3.5	0 to 8	0.07
Peripheral pulse (bpm)	74.5 (65–82.8)	70 (61–75.3)	-5	-11 to 1	0.09

BMI = body mass index; CI = confidence interval; DBP = diastolic blood pressure; IQR = interquartile range; SBP = systolic blood pressure; WC = waist circumference

*Mann–Whitney U test (Hodges–Lehmann median difference)

Procedural characteristics, including fluoroscopy time, radiation dose, and contrast volume, did not significantly differ between groups (Table 3).

**Table 3:** Comparison of procedural characteristics between groups

	Control Median (IQR)	Intervention Median (IQR)	Difference	95%CI	P*
FT (min)	1.9 (1.4–3.8)	1.7 (1–3.7)	-0.3	-0.9 to 0.3	0.28
DAP (uG/m^2^)	694.7 (461.7–1568.7)	660 (442.7–1410.3)	-35.13	-341.5 to 239.5	0.73
vol.CM (ml)	82 (67.3–100.3)	76 (65–93.8)	-2	-15 to 11	0.76

CI = confidence interval; DAP = dose-area product; FT = fluoroscopy time; IQR = interquartile range; vol.CM = contrast medium volume

*Mann–Whitney U test (Hodges–Lehmann median difference)

BIOCHEMICAL RESPONSE TO CORONARY ANGIOGRAPHY. At 24 h post-procedure, both groups demonstrated an increase in hs-troponin I, consistent with procedure-related myocardial stress (Table 4). Hs-CRP levels increased in both groups, with a trend toward a smaller rise in the intervention group (Supplemental Figure S1), although this difference was not statistically significant. After correction for multiple testing, hs-CRP remained significantly elevated, whereas changes in creatinine and eGFR did not retain statistical significance (Supplemental Table S3).

**Table 4:** Differences in biochemical markers between baseline and 24 h post-procedure in each patient group

	Baseline Median (IQR)	24 h post-procedure Median (IQR)	Difference	95%CI	P*
Control group					
hs-troponin I	3.3 (0.7–4.7)	4.4 (1.5–13.2)	1	0.25 to 5.6	0.006
Creatinine	75 (68–90)	74 (65–85)	-2.5	-6 to 0.5	0.06
eGFR	79.6 (67.6–90.9)	84.1 (75.9–93.1)	2.6	0 to 6.2	0.04
hs-CRP	1.5 (0.7–3.7)	2.1 (0.9–3.4)	0.2	-0.05 to 0.55	0.17
Intervention group					
hs-troponin I	3.7 (1.7–6.6)	5.1 (2.2–18.4)	1.6	0.4 to 15.1	0.002
Creatinine	74 (66–87)	79 (64–92)	2.5	-0.5 to 6.0	0.11
eGFR	80.7 (74.2–92.5)	78.2 (67.9–91.2)	-2.5	-5.6 to 0.4	0.12
hs-CRP	1.75 (0.9–2.8)	2.4 (1.2–3.9)	0.65	0.4 to 1.1	< 0.001

CI = confidence interval; eGFR = estimated glomerular filtration rate; hs-CRP = high-sensitivity C-reactive protein; IQR = interquartile range; 24 h post-procedure = 24 h post-coronary angiography

*Wilcoxon signed-rank test (Hodges-Lehmann median difference)

**Fig. 1:**
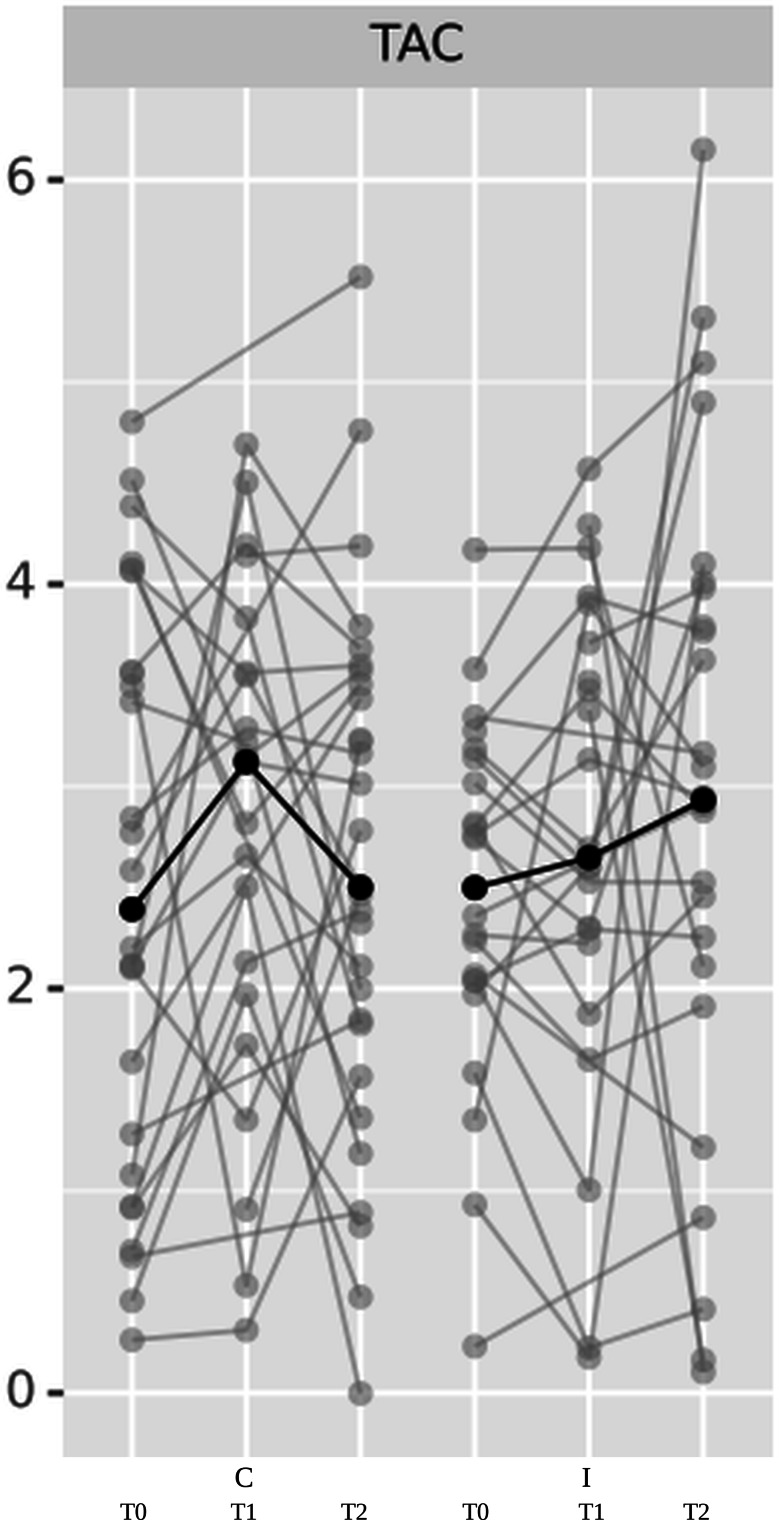
Total antioxidant capacity (TAC) Over Time in the Control (C) and Intervention (I) Groups. TAC was measured at baseline (T0), 4 h (T1), and 24 h (T2) after coronary angiography. Grey lines denote individual trajectories; black dots represent group medians. Values are expressed as µM uric acid equivalents. No statistically significant between-group differences were observed after multiple testing correction

DIFERENCES IN TOTAL ANTIOXIDANT CAPACITY. TAC values showed different temporal patterns between groups (Fig. 1). In the control group, TAC increased at 4 h (T1) and declined at 24 h (T2). In the intervention group, TAC values gradually increased from baseline to 24 h. At T2, median TAC values were numerically higher in the intervention group compared with the control group. However, between-group differences did not remain statistically significant after correction for multiple testing.

**Fig. 2:**
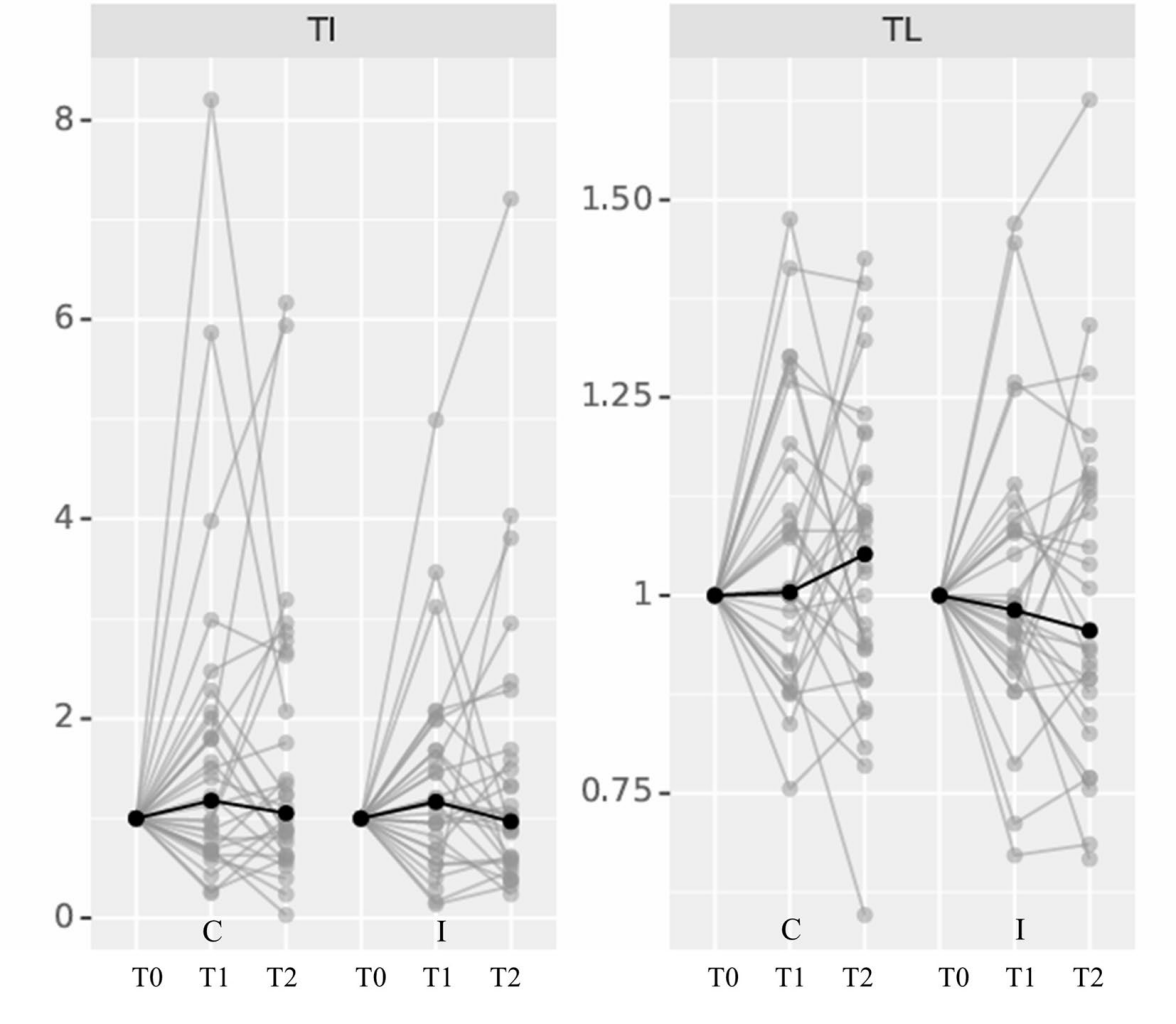
Oxidative DNA damage over time in the control (C) and intervention (I) groups. Normalized values of tail intensity (TI) and tail length (TL), assessed by alkaline comet assay, are presented at baseline (T0), 4 h (T1), and 24 h (T2) following coronary angiography. Grey lines represent individual participant trajectories, and black dots indicate group medians at each time point

OXIDATIVE DNA DAMAGE (ALKALINE COMET ASSAY). Between-group comparisons of raw comet assay parameters are presented in Supplemental Table S4, while longitudinal adjusted analyses are illustrated in Fig. 2. For tail intensity (TI), no significant baseline difference was observed between groups (T0; *p* = 0.067). At 24 h (T2), median TI was lower in the intervention group compared with the control group (*p* = 0.019). For tail length (TL), modest baseline differences were observed (T0; *p* = 0.033). At both 4 h (T1; *p* < 0.01) and 24 h (T2; *p* < 0.01), TL values were lower in the intervention group compared with controls. Longitudinal mixed-effects models demonstrated consistent directional effects favoring lower TI and TL values in the intervention group at 24 h (Fig. 2).

**Fig. 3:**
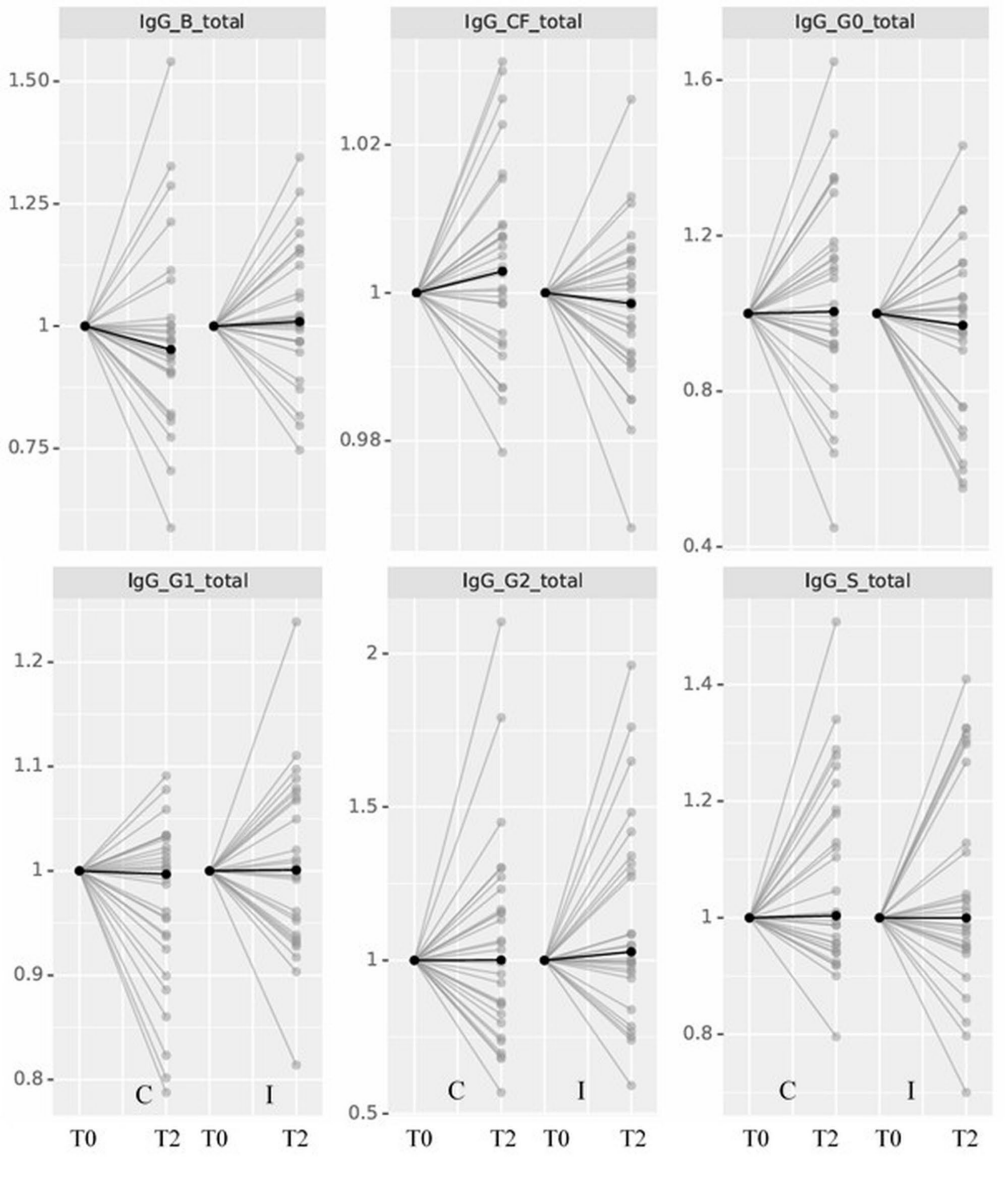
Changes in IgG N-Glycosylation Traits in the Control (C) and Intervention (I) Groups. Normalized values of derived IgG N-glycan traits are presented at baseline (T0) and 24 h (T2) following coronary angiography. Grey lines represent individual participant trajectories, and black dots indicate group medians at each time point. Displayed traits include bisecting GlcNAc (B), core fucosylation (CF), agalactosylation (G0), monogalactosylation (G1), digalactosylation (G2), and sialylation (S)

**Fig. 4:**
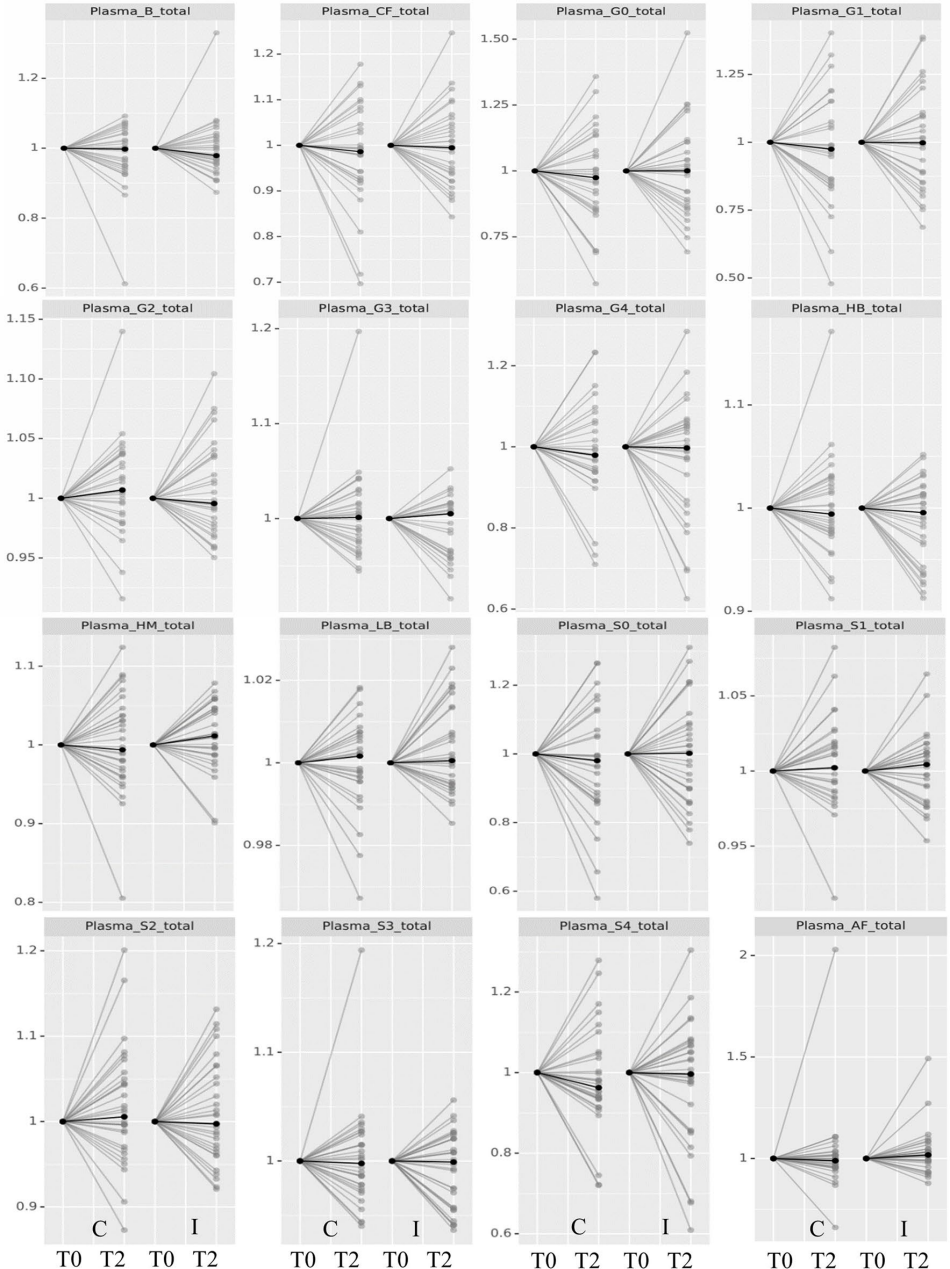
Plasma protein N-glycosylation changes in the control (C) and intervention (I) groups. Normalized values of derived plasma N-glycan traits for each participant at baseline (T0) and 24 h after coronary angiography (T2). Individual trajectories (grey lines) and group medians (black dots) are presented for each glycosylation feature: bisecting GlcNAc (B), core fucosylation (CF), agalactosylation (G0), monogalactosylation (G1), digalactosylation (G2), trigalactosylation (G3), tetragalactosylation (G4), highly branched glycans (HB), high-mannose glycans (HM), low-branched glycans (LB), asialylated glycans (S0), monosialylated glycans (S1), di-sialylated glycans (S2), trisialylated glycans (S3), tetrasialylated glycans (S4), and antennary fucosylation (AF)

CHANGES IN IMMUNOGLOBULIN G AND TOTAL PLASMA PROTEIN N-GLYCOSYLATION. A total of 22 derived glycan traits were analyzed, including 6 immunoglobulin G (IgG) and 16 total plasma protein traits (Figs. 3 and 4; Supplemental Tables S5-S6).

### IgG N-glycosylation

In the control group, 24 h after coronary angiography, relative increases were observed in agalactosylated (G0) and core-fucosylated (CF) IgG glycans, accompanied by reductions in galactosylated structures (G1, G2) (Fig. 3).

In contrast, the intervention group demonstrated decreases in core fucosylation (Effect = -0.026, SE = 0.014) and agalactosylation (Effect = -0.017, SE = 0.015), with corresponding increases in monogalactosylated (G1; Effect = 0.016, SE = 0.012) and digalactosylated (G2; Effect = 0.012, SE = 0.015) glycans (Supplemental Table S5). Bisecting GlcNAc (B) showed a small positive effect estimate (Effect = 0.022, SE = 0.014).

Although none of the individual IgG traits remained statistically significant after Benjamini–Hochberg correction, effect estimates were directionally consistent across related glycan features (Supplemental Table S5).

### Total plasma protein N-glycosylation

Mixed-effects analyses of total plasma protein glycans showed small effect sizes across traits (Fig. 4; Supplemental Table S6).

Positive effect estimates were observed for monogalactosylation (G1), agalactosylation (G0), low-branched glycans (LB), and asialylated glycans (S0), while negative effect estimates were observed for trigalactosylated (G3), highly branched (HB), and disialylated (S2) glycans.

No plasma protein glycan trait reached statistical significance before or after multiple testing correction (Supplemental Table S6).

## Discussion

The present randomized study suggests that a single pre-procedural dose of the SGLT2 inhibitor empagliflozin modulates oxidative and inflammatory responses following elective coronary angiography. Compared with standard care, the intervention group exhibited attenuation of oxidative DNA damage, differential temporal dynamics of total antioxidant capacity, and directional changes in IgG and total plasma protein N-glycosylation. The study was conducted as a prospective, randomized, double-blind interventional trial in accordance with established methodological principles for controlled study design [20]. The overall comparability of baseline characteristics between groups supports the internal validity of the study. Although baseline systolic blood pressure differed slightly between groups, the difference was modest and other baseline clinical characteristics were comparable, making a substantial influence on oxidative stress responses unlikely. Importantly, comparable procedural parameters, including fluoroscopy time, radiation dose, and contrast volume, make it unlikely that the observed biological differences were confounded by variations in procedural exposure. The sampling intervals were also aligned with the pharmacokinetic profile of empagliflozin, which reaches peak plasma concentrations within approximately 1–2 h after administration, allowing evaluation of early drug-related biological effects during the first hours following coronary angiography [21]. The simultaneous assessment of total antioxidant capacity, oxidative DNA damage, and glycosylation patterns enabled a multidimensional evaluation of redox and inflammatory responses, in accordance with current methodological recommendations for measuring oxidative stress in vivo [22]. Coronary angiography induced measurable myocardial and inflammatory activation, reflected by increased hs-troponin I and hs-CRP levels in both groups. These findings are consistent with previous reports showing that even diagnostic coronary procedures trigger systemic inflammatory responses [23, 24]. A trend toward a smaller increase in hs-CRP was observed in the intervention group, although this difference was not statistically significant, and is in line with prior clinical evidence demonstrating anti-inflammatory effects of SGLT2 inhibitors [25]. The transient creatinine rise and eGFR reduction observed after empagliflozin administration likely reflect hemodynamic adaptation mediated by tubuloglomerular feedback rather than acute kidney injury [26–28].

Empagliflozin was associated with a more stable trajectory of total antioxidant capacity, characterized by progressive stabilization at 24 h, whereas the control group exhibited a biphasic response with early elevation followed by decline. This pattern is consistent with the reported antioxidant and cardioprotective properties of SGLT2 inhibitors [29–31].

Mechanistic studies have demonstrated that SGLT2 inhibition attenuates oxidative stress through multiple pathways, including reduction of mitochondrial reactive oxygen species production [32], suppression of fibrosis-related TGF-β/SMAD signaling [33], and decreased intracellular and mitochondrial ROS in cardiac microvascular cells [34]. In human myocardium, SGLT2 inhibitors have been shown to reduce NADPH oxidase activity via the AMPK/Rac1 pathway and limit downstream inflammatory and apoptotic signaling [35]. Together, these mechanisms provide biological plausibility for the redox-modulating effects observed in the present study.

Oxidative DNA damage increased transiently following coronary angiography, with elevations in both tail intensity and tail length at 4 h in both groups. The magnitude of increase was numerically greater in the control group. By 24 h, both parameters declined toward baseline values, with lower levels observed in the intervention group. Although statistical significance was attenuated after multiple testing correction, the consistent directionality across comet assay parameters supports a potential attenuation of procedure-related oxidative DNA injury. These findings are compatible with experimental evidence indicating that SGLT2 inhibitors modulate redox-sensitive and inflammatory signaling pathways relevant to cardiovascular protection [36].

Directional changes in IgG and total plasma protein glycosylation were observed following empagliflozin administration, including relative reductions in agalactosylated IgG glycans, commonly associated with pro-inflammatory activity, and relative preservation of galactosylated structures linked to anti-inflammatory profiles [37]. Alterations in IgG and plasma protein N-glycosylation have previously been linked to inflammatory and immune modulation in cardiovascular conditions [38]. These findings are biologically plausible given the established anti-inflammatory effects of SGLT2 inhibitors, including suppression of NF-κB signaling and downstream cytokine expression such as IL-6 and TNF-α [39], as well as reductions in inflammatory markers and endothelial activation [40].

Among the IgG-derived traits, the most consistent directional change was a decrease in core fucosylation. Core fucose plays a regulatory role in Fcγ receptor interactions and antibody effector functions [41, 42], and shifts in this modification may influence inflammatory signaling. A directional increase in bisecting GlcNAc was observed, a modification implicated in modulation of IgG effector function and inflammatory signaling [43]. However, given the lack of statistical significance after correction for multiple testing, these findings should be interpreted as exploratory and hypothesis-generating. Although individual glycan traits did not retain statistical significance after correction for multiple testing, the consistent directional pattern across related structures supports biological plausibility.

Glycosylation of total plasma proteins plays a regulatory role in protein stability and function. In the present study, modest directional shifts were observed following empagliflozin administration, suggesting potential modulation of plasma N-glycosylation patterns. A decrease in highly branched glycans and a relative increase in bisecting GlcNAc were noted, glycan features previously linked to inflammatory and immune regulation [44].

Changes in galactosylation traits included relative increases in agalactosylated and monogalactosylated glycans within the intervention group, accompanied by reductions in more extensively galactosylated structures. Similar remodeling of plasma protein glycosylation has been described in inflammatory and metabolic conditions, including diabetes and atherosclerosis [45].

Alterations in sialylation were characterized by relative increases in asialylated glycans and decreases in more highly sialylated species, without changes in tetrasialylated structures. An increase in antennary fucosylation was also observed; this modification contributes to the synthesis of sialyl-Lewis X epitopes, which participate in selectin-mediated immune interactions [46, 47].

Notably, reductions in trigalactosylated and highly branched glycans, structures commonly enriched in acute-phase proteins [48], were detected predominantly in the intervention group. Although these findings did not retain statistical significance after correction for multiple testing, the coordinated pattern across multiple glycan features may suggests a structured remodeling rather than random fluctuation.

### Study limitations

Several limitations should be acknowledged. First, the relatively small sample size may have limited statistical power to detect modest biomarker effects. Second, exclusion of patients with diabetes restricts generalizability to populations characterized by higher baseline oxidative stress. Third, administration of a single pre-procedural dose of empagliflozin may not fully capture the drug’s cumulative antioxidant and anti-inflammatory effects, which are likely to evolve with sustained exposure. Finally, the absence of follow-up beyond 24 h precludes assessment of delayed or longer-term redox and inflammatory responses.

## Conclusions

In this randomized controlled study, pre-procedural empagliflozin administration was associated with attenuation of oxidative DNA damage and differential modulation of antioxidant and glycosylation biomarkers following coronary angiography. Directional changes in IgG N-glycosylation and inflammatory markers further suggest potential immunomodulatory effects. Although several findings did not retain statistical significance after multiple testing correction, the consistency of biological signals supports a possible peri-procedural cytoprotective effect that warrants confirmation in larger and longer-term studies.

## Clinical perspectives

COMPETENCY IN MEDICAL KNOWLEDGE AND PATIENT CARE: Coronary angiography induces transient oxidative and inflammatory responses that may contribute to vascular and renal stress. In this randomized study, pre-procedural administration of empagliflozin was associated with attenuation of oxidative DNA damage and modulation of antioxidant and glycosylation biomarkers.

TRANSLATIONAL OUTLOOK: These findings support further investigation of SGLT2 inhibition as a potential peri-procedural cytoprotective strategy in invasive cardiology. Larger studies are needed to determine whether modulation of redox and glycosylation pathways translates into clinically meaningful benefit.

## Supplementary Information

Below is the link to the electronic supplementary material.

Supplementary Material 1

## Data Availability

The datasets used and/or analysed during the study are available from the corresponding author on reasonable request.

## Abbreviations

ACS: Acute coronary syndrome
ECG: Electrocardiogram
eGFR: Estimated glomerular filtration rate
FDR: False discovery rate
HFrEF: Heart failure with reduced ejection fraction
IgG: Immunoglobulin G
PCI: Percutaneous coronary intervention
ROS: Reactive oxygen species
SGLT2i: Sodium-glucose cotransporter 2 inhibitors
TAC: Total antioxidant capacity
TI: Tail intensity
TL: Tail length
