# Supplementary material for: Acute vascular redox modulation by SGLT2 inhibition in non-diabetic patients
Source: Cardiovasc Diabetol Endocrinol Rep. 2026 Jun 18;12:34. doi: 10.1186/s40842-026-00304-5 (PMC13277242; doi:10.1186/s40842-026-00304-5)
Supplement: Supplementary file 1 — Supplementary Material 1 [file 40842_2026_304_MOESM1_ESM.docx]

Acute vascular redox modulation by SGLT2 inhibition in non-diabetic patients: Randomized trial

Brief title: Antioxidative Role of SGLT2 Inhibitors in Angiography

Authors:

Katica Cvitkusic Lukenda, MD, PhD a, b

Ana Cipak Gasparovic, PhD c

Mirta Milic, PhD d

Barbara Radovani, PhD e, f

Frano Vučković, PhD f

Jelena Jakab, MD, PhD b

Domagoj Vucic, MD a

Ana Kovacevic, MD a

Ivan Gudelj, PhD e, f

Departments and institutions:

a Department of Cardiology, General Hospital Dr. Josip Bencevic, A. Stampara 42, HR-35000 Slavonski Brod, Croatia

b Faculty of Dental Medicine and Health Osijek, Josip Juraj Strossmayer University of Osijek, Osijek, Croatia

c Rudjer Boskovic Institute, Zagreb

d Institute for Medical Research and Occupational Health, Zagreb

e Faculty of Biotechnology and Drug Development, University of Rijeka, Rijeka

f Genos Glycoscience Research Laboratory, Zagreb

Address for correspondence:

Katica Cvitkusic Lukenda, Department of Cardiology, General Hospital Dr. Josip Bencevic, A. Stampara 42, HR-35000 Slavonski Brod, Croatia

Telephone number: +385 98 556 576

Fax number: +385 35 201 700

e-mail address: kclukenda@gmail.com

Supplementary materials

Supplementary methods

ETHICAL APPROVALS. Ethical approval was obtained from the Ethics Committee of the General Hospital “Dr. Josip Benčević” in Slavonski Brod (Approval No. 04000000/23-65), the Ethics Committee of the Institute for Medical Research and Occupational Health in Zagreb (Approval No. 100-21/23-12), and the Bioethics Committee of the Ruđer Bošković Institute in Zagreb (Approval No. BEP-5968/1-2023).

STATISTICAL ANALYSIS. Data distribution was assessed using the Shapiro-Wilk test. Normally distributed variables are shown as mean ± SD, and non-normally distributed variables as median (IQR). Between-group comparisons were performed using Student’s t-test or the Mann-Whitney U test. Categorical variables were compared using the χ² test. Changes before and after coronary angiography were evaluated with 2-way ANOVA. Correlations were analyzed with Pearson’s or Spearman’s test, depending on the variable type. Regression models estimated the influence of predictors on oxidative stress, with statistical significance set at p < 0.05.

For glycan analysis, UHPLC data were normalized to total area and batch-corrected using the ComBat method after log transformation. Technical variation (plate ID) was modeled as a covariate. Longitudinal changes in glycans, comet assay parameters (tail intensity, tail length), and biochemical markers (e.g., hs-troponin I, creatinine, eGFR, hs-CRP) were assessed using linear mixed-effects models, adjusting for the effect of empagliflozin. Variables were normalized using inverse rank-based transformation to allow effect size comparability. FDR was controlled at 0.05 using the Benjamini-Hochberg method. Analyses were conducted in R (v4.3.3).

Comet assay results were summarized as mean, median, range, SD, and SE, and analyzed using ANOVA, followed by Scheffé’s post hoc test for multiple comparisons. The experiment included internal controls and was performed in duplicate to ensure consistency between replicates. No differences were observed when comparing two gels from the same sample; therefore, values from both gels were combined and analyzed as a single sample of 100 comets.

Online figure

Supplemental Figure 1. Trend of hs-CRP Levels After Coronary Angiography


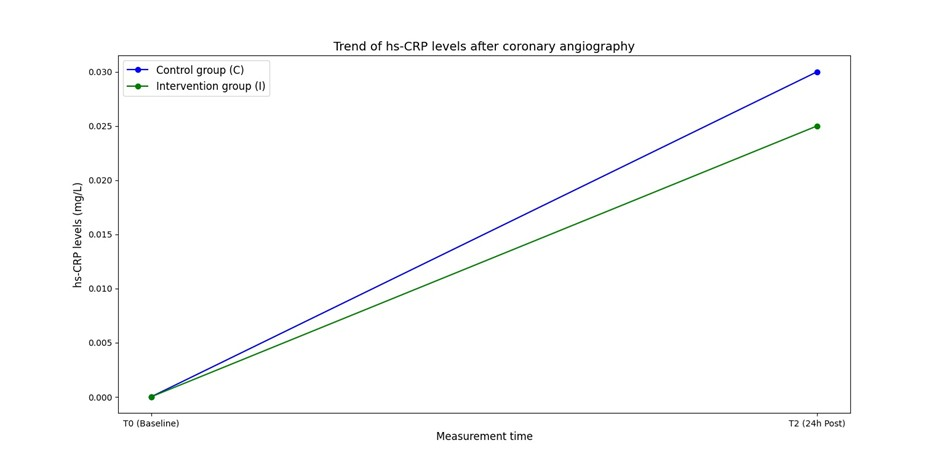


The figure presents changes in hs-CRP levels at baseline (T0) and 24 hours after coronary angiography (T2) are presented for the control (blue line, circles) and intervention groups (green line, triangles). Both groups showed an increase in hs-CRP levels over time; however, the magnitude of increase was smaller in the intervention group compared to the control group (Python 3.12.8).

Supplementary tables

Supplemental Table 1. The mathematical expressions for each derived N-glycan trait

| **IgG N-glycan traits** | |
| --- | --- |
| Agalactosylation (G0) | GP1 + GP2 + GP3 + GP4 + GP6 |
| Monogalactosylation (G1) | GP7 + GP8 + GP9 + GP10 + GP11 |
| Digalactosylation (G2) | GP12 + GP13 + GP14 + GP15 |
| Bisecting GlcNAc (B) | GP3 + GP6 + GP10 + GP11 + GP13 + GP15 + GP19 + GP22 + GP24 |
| Core fucosylation (CF) | GP1 + GP4 + GP6 + GP8 + GP9 + GP10 + GP11 + GP14 + GP15 + GP16 + GP18 + GP19 + GP20 + GP23 + GP24 |
| Sialylation (S) | GP16 + GP17 + GP18 + GP19 + GP20 + GP21 + GP22 + GP23 + GP24 |
| **Plasma N-glycan traits** | |
| Low-branched (LB) | GP1 + GP2 + GP3 + GP4 + GP5 + GP6 + GP8 + GP9 + GP10 + GP11 + GP13 + GP14 + GP15 + GP16 + GP17 + GP18 + GP20 + GP21 + GP22 + GP23 |
| Highly-branched (HB) | GP24 + GP25 + GP26 + GP27 + GP28 + GP29 + GP30 + GP31 + GP32 + GP33 + GP34 + GP35 + GP36 + GP37 + GP38 + GP39 |
| Agalactosylation (G0) | GP1 + GP2 |
| Monogalactosylation (G1) | GP3 + GP4 + GP5 + GP6 + GP13 |
| Digalactosylation (G2) | GP8 + GP9 + GP10 + GP11 + GP14 + GP15 + GP16 + GP17 + GP18 + GP20 + GP21 + GP22 + GP23 |
| Trigalactosylation (G3) | GP24 + GP25 + GP26 + GP27 + GP28 + GP29 + GP30 + GP31 + GP32 + GP33 + GP34 + GP35 |
| Tetragalactosylation (G4) | GP36 + GP37 + GP38 + GP39 |
| Asialylation (S0) | GP1 + GP2 + GP3 + GP4 + GP5 + GP6 + GP8 + GP9 + GP10 + GP11 |
| Monosialylation (S1) | GP13 + GP14 + GP15 + GP16 + GP17 |
| Disialylation (S2) | GP18 + GP20 + GP21 + GP22 + GP23 + GP24 + GP25 + GP26 + GP27 |
| Trisialylation (S3) | GP28 + GP29 + GP30 + GP31 + GP32 + GP33 + GP34 + GP35 + GP36 |
| Tetrasialylation (S4) | GP37 + GP38 + GP39 |
| Bisecting GlcNAc (B) | GP2 + GP3 + GP6 + GP9 + GP11 + GP15 + GP17 + GP23 |
| Antenary fucosylation (AF) | GP27 + GP33 + GP35 + GP39 |
| Core fucosylation (CF) | GP1 + GP2 + GP4 + GP5 + GP6 + GP10 + GP11 + GP13 + GP16 + GP17 + GP22 + GP23 + GP31 + GP34 + GP35 |
| High-mannose (HM) | GP7 + GP12 + GP19 |

GP = glycan peak

Supplemental Table 2. Baseline Laboratory Characteristics of Control and Intervention Groups

|  | Control Median (IQR) | Intervention Median (IQR) | Difference | 95%CI | *P** |
| --- | --- | --- | --- | --- | --- |
| Erythrocytes | 4.6 (4.3 – 4.9) | 4.6 (4.3 – 4.8) | -0.02 | -0.24 to 0.20 | 0.88 |
| Hemoglobin | 136.5  (124.3 – 147.5) | 138  (130.5 – 145.5) | 1.0 | -6 to 9 | 0.76 |
| Hematocrit | 41.2 (37.6 - 44) | 40.5 (39 – 43.1) | -0.3 | -2.2 to 1.8 | 0.81 |
| Leukocytes | 6.7 (5.2 – 7.8) | 6.1 (5.4 - 8) | 0.07 | -0.96 to 0.85 | 0.91 |
| Platelets | 195.5  (168.8 - 236) | 216.5  (196.5 – 258.8) | 24 | -1 to 49 | 0.06 |
| BG | 5.5 (4.9 – 5.8) | 5.6 (5.2 – 6.3) | 0.2 | -0.1 to 0.6 | 0.21 |
| Urea | 5.9 (5.3 – 7.4) | 5.8 (4.7 – 6.9) | -0.3 | -1.1 to 0.5 | 0.48 |
| Potassium | 4.2 (4 – 4.4) | 4.3 (4.1 – 4.5) | 0.1 | -0.1 to 0.3 | 0.23 |
| Sodium | 141 (140 - 142) | 141 (139 - 142) | 0 | -1 to 0 | 0.32 |
| Chloride | 105  (102.8 - 106) | 104.5  (102 - 106) | 0 | -2 to 1 | 0.59 |
| Bilirubin | 11 (8 - 18) | 11 (8 - 14) | -1 | -4 to 2 | 0.56 |
| AST | 24 (22 – 29.5) | 28 (22 – 32.3) | 2 | -1 to 6 | 0.18 |
| ALT | 24 (17.8 – 26.5) | 27 (19.8 – 38.3) | 4 | -1 to 10 | 0.09 |
| AF | 77.5 (59 - 86) | 73.5 (59.8 - 90) | 0 | -12 to 11 | 0.97 |
| GGT | 19 (15 – 29.3) | 22 (17.3 - 31) | 2 | -2 to 8 | 0.23 |
| Cholesterol | 4.5 (3.7 – 5.6) | 4.8 (4 – 5.3) | 0.2 | -0.5 to 0.8 | 0.61 |
| Triglyceride | 0.9 (0.8 – 1.3) | 1.2 (0.9 – 1.7) | 0.2 | -0.1 to 0.5 | 0.15 |
| HDL-C | 1.3 (1.1 – 1.6) | 1.3 (1.2 – 1.6) | 0.1 | -0.1 to 0.2 | 0.26 |
| LDL-C | 2.7 (2.2 – 3.6) | 3 (2.5 – 3.4) | 0.2 | -0.3 to 0.6 | 0.54 |
| Iron | 17.5 (10 - 22) | 18 (12 - 21) | 1 | -2 to 4 | 0.42 |
| UIBC | 43 (37.3 – 51.3) | 39 (34.8 – 47.5) | -2 | -8 to 4 | 0.44 |
| TIBC | 61 (53 - 65) | 59 (54.5 – 64.3) | 0 | -5 to 4 | 0.91 |
| Ferritin | 114 (43.4 – 179.6) | 145.1(78.6 – 278.7) | 31.9 | -13.9 to 93.4 | 0.17 |
| HbA1C | 36 (34 – 39.3) | 38 (36 – 39.3) | 1 | -1 to 3 | 0.20 |
| HbA1C % | 5.5 (5.3 – 5.7) | 5.6 (5.4 – 5.7) | 0.10 | -0.10 to 0.30 | 0.23 |
| Vitamin D | 57.9  (41.1 – 85.1) | 55.7  (40.8 – 69.4) | -5.9 | -18.9 to 8.0 | 0.43 |
| PTH | 46(36.2 – 60.2) | 44.3 (39.2 – 54.5) | -0.99 | -8.6 to 6.6 | 0.87 |
| TSH | 1.6 (1 – 2.4) | 1.8 (1.1 – 2.4) | 0.07 | -0.42 to 0.51 | 0.80 |
| Total Ca | 2.3 (2.3 – 2.4) | 2.4 (2.3 – 2.5) | 0.03 | -0.03 to 0.08 | 0.32 |
| Ionized Ca | 1.2 (1.2 – 1.2) | 1.2 (1.2 – 1.2) | 0.01 | -0.01 to 0.03 | 0.32 |
| Phosphates | 1 (0.9 – 1.1) | 1 (0.9 – 1.1) | 0 | -0.09 to 0.08 | 0.95 |

BG = blood glucose; AF = alkaline phosphatase; ALT = alanine aminotransferase; AST = aspartate aminotransferase; GGT = gamma-glutamyl transferase; HbA1C = glycated hemoglobin; HDL-C = high-density lipoprotein cholesterol; LDL-C = low-density lipoprotein cholesterol; PTH = parathyroid hormone; TIBC = total iron-binding capacity; TSH = thyroid-stimulating hormone; UIBC = unsaturated iron-binding capacity.

*Mann-Whitney U test (Hodges-Lehmann median difference)

Supplemental Table 3. Effect Estimates, Standard Errors, and Adjusted p-values for Biochemical Markers

|  | Efekt | SP | *p* | *p_adj** |  |
| --- | --- | --- | --- | --- | --- |
| hs Troponin I | 0.002 | 0.008 | 0.82232 | 0.95285 |  |
| Creatinine | 0.015 | 0.006 | 0.01015 | 0.24701 |  |
| eGFR | -0.017 | 0.006 | 0.00417 | 0.15222 |  |
| hs CRP | 0.014 | 0.004 | 0.00059 | 0.04313 |  |

Effect estimates represent the impact of the intervention on biochemical marker levels. Standard errors (SE) and Benjamini–Hochberg adjusted p-values (p_adj) are provided to control for multiple testing.

*Benjamini-Hochberg correction

Supplemental Table 4. DNA Damage Measured by Tail Intensity (TI) and Tail Length (TL) in the Comet Assay

|  | Intervention Median (IQR) | Control Median (IQR) | *P** |
| --- | --- | --- | --- |
| Tail Intensity (TI) |  |  |  |
| T0, n= 3000 | 4.59 (0.78-12.46) | 4.16 (0.66-12.09) | 0.067 |
| T1, n= 3000 | 4.91 (0.94-13) | 5.44 (0.89-14.92) | 0.057 |
| T2, n= 3000 | 4.52 (0.68-12.58) | 4.75 (0.73-14.58) | 0.019 |
| Tail Length (TL) | | | |
| T0, n= 3000 | 22.08 (17.92-17.92) | 22.5 (18.33-18.33) | 0.033 |
| T1, n= 3000 | 22.5 (17.5 -27.92) | 22.92 (18.33-29.58) | <0.01 |
| T2, n= 3000 | 22.08 (17.08-27.5) | 22.92 (17.92-29.17) | <0.01 |

DNA damage was assessed using the comet assay, with tail intensity and tail length reflecting the extent of single-strand DNA breaks. Data are presented as median values with interquartile ranges.

*Mann-Whitney U test

Supplemental Table 5. Effect of the Intervention on the IgG N-Glycan Profile

|  | Effect | SE | *p* | *p*_adj* |
| --- | --- | --- | --- | --- |
| IgG_B_total | 0.022 | 0.014 | 0.11963 | 0.81575 |
| IgG_CF_total | -0.026 | 0.014 | 0.06124 | 0.81575 |
| IgG_G0_total | -0.017 | 0.015 | 0.27118 | 0.81575 |
| IgG_G1_total | 0.016 | 0.012 | 0.21015 | 0.81575 |
| IgG_G2_total | 0.012 | 0.015 | 0.42976 | 0.81575 |
| IgG_S_total | -0.006 | 0.014 | 0.68936 | 0.86933 |

Effect estimates represent the impact of the intervention on IgG N-glycan profiles. Standard errors (SE) and Benjamini–Hochberg adjusted p-values (p_adj) are provided to control for multiple testing.

*Benjamini-Hochberg correction

Supplemental Table 6. Effect of the Intervention on the Total Plasma Protein N-Glycans

|  | Effect | SE | *p* | *p*_adj* |
| --- | --- | --- | --- | --- |
| Plasma_B_total | 0.003 | 0.006 | 0.63470 | 0.86695 |
| Plasma_CF_total | 0.004 | 0.009 | 0.62670 | 0.86695 |
| Plasma_G0_total | 0.007 | 0.009 | 0.39911 | 0.81575 |
| Plasma_G1_total | 0.009 | 0.011 | 0.39788 | 0.81575 |
| Plasma_G2_total | -0.005 | 0.011 | 0.64749 | 0.86695 |
| Plasma_G3_total | -0.007 | 0.005 | 0.14190 | 0.81575 |
| Plasma_G4_total | -0.001 | 0.009 | 0.95639 | 0.99738 |
| Plasma_HB_total | -0.007 | 0.005 | 0.18506 | 0.81575 |
| Plasma_HM_total | 0.002 | 0.007 | 0.77639 | 0.92913 |
| Plasma_LB_total | 0.005 | 0.005 | 0.31348 | 0.81575 |
| Plasma_S0_total | 0.010 | 0.011 | 0.35418 | 0.81575 |
| Plasma_S1_total | -0.002 | 0.005 | 0.66712 | 0.86933 |
| Plasma_S2_total | -0.007 | 0.012 | 0.59213 | 0.86451 |
| Plasma_S3_total | -0.005 | 0.005 | 0.27666 | 0.81575 |
| Plasma_S4_total | 0.000 | 0.009 | 0.99757 | 0.99757 |
| Plasma_AF_total | 0.006 | 0.005 | 0.26605 | 0.81575 |

Effect estimates represent the impact of the intervention on total plasma protein N-glycan profiles. Standard errors (SE) and Benjamini–Hochberg adjusted p-values (p_adj) are provided to control for multiple testing.

*Benjamini-Hochberg correction
